# Molecular and Immunogenic Properties of Apyrase SP01B and D7-Related SP04 Recombinant Salivary Proteins of *Phlebotomus perniciosus* from Madrid, Spain

**DOI:** 10.1155/2013/526069

**Published:** 2013-09-22

**Authors:** Inés Martín-Martín, Ricardo Molina, Maribel Jiménez

**Affiliations:** Unidad de Entomología Médica, Servicio de Parasitología, Centro Nacional de Microbiología, Instituto de Salud Carlos III, Carretera Majadahonda-Pozuelo s/n, Majadahonda, 28220 Madrid, Spain

## Abstract

Sand fly salivary proteins are on the spotlight to become vaccine candidates against leishmaniasis and to markers of exposure to sand fly bites due to the host immune responses they elicit. Working with the whole salivary homogenate entails serious drawbacks such as the need for maintaining sand fly colonies and the laborious task of glands dissection. In order to overcome these difficulties, producing recombinant proteins of different vectors has become a major task. In this study, a cDNA library was constructed with the salivary glands of *Phlebotomus perniciosus* from Madrid, Spain, the most widespread vector of *Leishmania infantum* in the Mediterranean basin. Analysis of the cDNA sequences showed several polymorphisms among the previously described salivary transcripts. The apyrase SP01B and the D7-related protein SP04 were successfully cloned, expressed in *Escherichia coli*, and purified. Besides, recombinant proteins were recognized by sera of hamsters and mice previously immunized with saliva through the exposure to uninfected sand fly bites. These results suggest that these two recombinant proteins conserved their immunogenic properties after expression in a prokaryote system. Therefore, this work contributes to expand the knowledge of *P. perniciosus* saliva that would be eventually used for the development of tools for vector control programs.

## 1. Introduction

Leishmaniasis is still one of the most important vector-borne diseases in terms of incidence as two million people per year are affected worldwide [1]. The causative agent of the aforementioned disease is *Leishmania* spp., a flagellate parasite which is transmitted by infected phlebotomine sand flies (Diptera: Psychodidae) during blood feeding. Arthropod saliva is actively involved in the transmission of pathogens to its host as it contains a complex cocktail of antihaemostatic and immunomodulatory molecules that are inoculated into the host skin during blood feeding of both infected and noninfected sand flies [2]. Concretely, sand fly salivary components are known to play an important role in the establishment of *Leishmania* spp. infection [3]. In the last years, research on sand fly salivary proteins has greatly increased, suggesting that salivary proteins could be successfully assayed both as anti-*Leishmania* vaccine candidates and as markers of exposure to sand flies [4, 5]. As hosts are bitten, they develop both humoral and cellular responses against sand fly saliva [6]. Moreover, a positive correlation has been observed between the number of bites and antibody levels [4, 7, 8]. Therefore, host exposure to sand flies can be measured by evaluating humoral responses against salivary antigens. This methodology is being applied by mainly using salivary gland extracts [4, 9–12]. Recombinant salivary proteins have already been produced for sand fly species such as *Lutzomyia longipalpis *and* Phlebotomus papatasi *[4, 8, 10, 11, 13, 14]. Some of these proteins have been already proved as good markers of exposure [11, 14]. However, since salivary proteins display high specificity, it is necessary to produce immunogenic salivary proteins for other sand fly species. 

In the western Mediterranean basin,* Leishmania infantum* is mainly transmitted by *Phlebotomus perniciosus *in an anthropozoonotic cycle where dogs have been traditionally considered the main reservoir [1]. However, other potential reservoirs such as hares (*Lepus granatensis*) have been recently involved in *L. infantum* transmission at a human leishmaniasis outbreak in Madrid [15, 16]. As a part of a management plan to control the disease in this environment, measuring exposure of reservoirs to the main vector involved, *P. perniciosus,* through the detection of anti-saliva antibodies, would be useful to evaluate whether actions taken to reduce leishmaniasis have been effective as previously done in India and Nepal [9].

In previous studies, our group described immunogenic salivary proteins of *P. perniciosus *and* P. argentipes. *These proteins were identified through the combination of two-dimensional electrophoresis and Western blot with sera of mice and hamsters experimentally exposed to uninfected sand flies [17, 18]. Therefore, in this study, our aim was to obtain some of the *P. perniciosus* salivary immunogenic molecules as recombinant proteins, through a cDNA library from salivary glands, from *P. perniciosus* and subsequent studies of the immunogenicity of the purified salivary proteins. Moreover, several polymorphisms between transcripts of the *P. perniciosus* cDNA from Madrid were compared to the previously annotated ones that belonged to specimens from Italy [19]. 

## 2. Material and Methods

### 2.1. Sand Flies and Salivary Glands Collection

*P. perniciosus* sand flies were maintained at 27°C and 17 : 7 light-darkness photoperiod at the Medical Entomology Unit of the Instituto de Salud Carlos III (ISCIII), Madrid, Spain. This colony was established in 1987 from sand flies captured at a leishmaniasis endemic area of Madrid [20]. Salivary glands from recently emerged up to 1-day-old sand flies were dissected and stored in RNA*later* (Invitrogen, San Diego, CA).

### 2.2. Salivary Gland cDNA Library Construction

A cDNA library was constructed with mRNA isolated from 165 salivary glands using the Micro-FastTrack mRNA isolation kit (Invitrogen, San Diego, CA). After isolation, mRNA was reverse transcrbted to cDNA and subsequently amplified by PCR following the instructions of the SMART cDNA library construction kit (Clontech). cDNAs were then fractionated by column chromatography before cloning. Directional cloning into *λ*TriplEx2 vector (Clontech) was achieved through *Sfi*IAB flanking sites incorporated by PCR during the cDNA amplification. The pool of cDNAs cloned into *λ*TriplEx2 vector was packaged into phage particles following the manufacturer's instructions (Gigapack III Gold Packaging Extract, Agilent). The resulting cDNA library was amplified following general molecular biology protocols [21]. 

### 2.3. cDNA Library Screening, Sequencing, and Bioinformatics

After plating the library by infecting log phase XL1-blue cells (Clontech) in the presence of *β*-D-thiogalactoside (IPTG) and 5-bromo-4-chloro-indolyl-*β*-D-galactopyranoside (X-Gal), white plaques were randomly picked from the agar plates and analyzed by PCR using *λ*TriplEx2 primers (forward: 5′-TCCGAGATCTGGACGAGC-3′ and reverse: 5′-TAATACGACTCACTATAGGGC-3′). Recombinant phages which presented the greatest insert sizes were converted into pTriplEx2 plasmid through Cre recombinase-mediated site-specific recombination. Plasmid DNA was then isolated and sequenced using the primer 5′*λ*TriplEx LD: 5′-CTCGGGAAGCGCGCCATTGTGTTGGT-3′ and the BigDye Terminator v3.1 Cycle Sequencing kit (PE Biosystems, Foster City, CA) in an ABI PRISM 3730XL DNA Analyzer (Applied Biosystems). Both strains of clones of interest were subsequently sequenced with reverse primer 3′*λ*TriplEx LD: 5′-ATACGACTCACTATAGGGCGAATTGGCC-3′. DNA electropherograms were manually inspected and corrected using Chromas program (McCarthy, Queensland, Australia). Sequence identities were determined by BLAST (http://blast.ncbi.nlm.nih.gov/). DNASTAR software (Lasergene, Madison, WI) was used for nucleotidic alignments (SeqMan program), and protein features were assigned by Protean tools. Amino acid sequences were aligned with ClustalW (http://www.ch.embnet.org/software/ClustalW.html) and refined using Boxshade server (http://www.ch.embnet.org/software/BOX_form.html). Glycosylation sites were predicted using NetNGlyc 1.0 Server (http://www.cbs.dtu.dk/services/NetNGlyc/) and NetOGlyc 3.1 Server (http://www.cbs.dtu.dk/services/NetOGlyc/) for N- and O-glycosylation sites, respectively [22, 23]. Phosphorylation sites prediction was done using NetPhos 2.0 Server (http://www.cbs.dtu.dk/services/NetPhos/) [24]. 

### 2.4. Cloning of Salivary Gland cDNAs

Salivary coding sequences of the apyrase SP01B and the D7-related protein SP04 were amplified by PCR from the corresponding pTriplEx2 plasmids using specific primers. cDNAs were cloned into PCR4-TOPO plasmid and transformed into TOP10 competent cells (Invitrogen, San Diego, CA). Restriction sites were incorporated by PCR from the recombinant PCR4-TOPO vector. *Sal*I (forward and reverse) was used as a restriction site (represented as bold letters): SP01B: 5′-**GTCGAC**ATGATATTGTTGAAATTG-3′ and 5′-**GTCGAC**TTACTTAATTCCTTTGGG-3′ and SP04: 5′-GAGAGA**GTCGAC**ATGAATACCTTATTG-3′ and 5′-GAGAGA**GTCGAC**CTAATAATTTGTTAATG-3′. The primers were manually designed according to the sequences obtained. All primers were synthesized at the Genomic Unit of ISCIII. The high-fidelity polymerase *Pfu *Turbo Hotstart (Stratagene, La Jolla, CA) was used in order to avoid point mutations. Coding sequences were excised from the plasmid by enzymatic digestion and ligated into pqE31 vector (Qiagen, Hilden, Germany). The recombinant expression vectors pqE31 were transformed into competent M15 (Qiagen, Hilden, Germany). All construction steps were verified by sequencing. 

### 2.5. Expression and Purification of Recombinant Salivary Proteins

Different expression and purification conditions were tested in order to optimize the processes for these specific proteins. Cultures of M15 cells that contained the pqE31 recombinant plasmid for SP01B and SP04 were grown in Luria-Bertani medium containing ampicillin (100 *μ*g/mL). Protein expression was induced by the addition of IPTG to a final concentration of 1 mM, and cultures were grown at 37°C for 3 hours. Bacterial cells were collected by centrifugation and lysed in 6 M guanidine hydrochloride, 100 mM NaH_2_PO_4_, 10 mM Tris-HCl, and pH 8. Cell debris was then separated by centrifugation, and the resultant supernatant was submitted to affinity chromatography by using Ni-NTA Superflow resin in prepacked 5 mL columns. Proteins were purified under denaturing conditions following the manufacturer's instructions (Qiagen, Hilden, Germany). Protein refolding was done by removing urea through dialysis against PBS (SnakeSkin Dialysis Tubing 10 kDa MWCO, Thermo Scientific, Goettingen, Germany). Proteins were then concentrated using the stirred ultrafiltration cell with 10 kDa MWCO membranes (Millipore, Bedford, MA) and quantified by gel in comparison with BSA standards. 

### 2.6. Western Blotting

Western blots were performed following standard protocols. Briefly, 1 *μ*g of recombinant protein was separated by SDS-PAGE and electroblotted onto a PVDF membrane which was incubated overnight in blocking buffer (3% BSA, Sigma, St. Louis, CA; 2% ECL Blocking reagent, Amersham, Piscataway, NJ). After washing, membranes were incubated for 2 hours with pooled sera (1 : 25) of either hamsters that were immunized against *P. perniciosus* saliva by the bite of uninfected sand flies through the exposure to 100 sand flies on a weekly schedule over 10 weeks [17] or mice exposed 13 times to 150 *P. perniciosus *(unpublished). Goat anti-hamster IgG and goat anti-mouse IgG peroxidase-conjugated antibodies (1 : 3500, Southern Biotech, Birmingham, AL and 1 : 500, AbD Serotec, resp.) were used, and immunogenic proteins were visualized by CN/DAB reagent following the manufacturer's instructions (Thermo Scientific, Goettingen, Germany). Experiments with sera of immunized and nonimmunized animals were carried out in parallel. For His-tag detection, mouse anti-RGS-His antibodies (1 : 3000, Qiagen, Hilden, Germany) were used in combination with the goat anti-mouse IgG peroxidase-conjugated antibodies (AbD Serotec).

## 3. Results and Discussion

### 3.1. cDNA Library Analysis

The expression library showed a titer of 3.6 × 10^6^ and 17.6% of nonrecombinant clones. The amplified library displayed a larger number of independent clones (1.2 × 10^12^) and a low number of nonrecombinant clones (1.92%). Therefore, this amplified library was used for subsequent analysis and cloning processes. 201 white plaques were randomly picked from IPTG/X-Gal plates and analyzed by PCR. 36 out of 201 plaques showing the greatest insert size were selected, converted into pTriplEx2, and further sequenced. Among the 25 complete transcripts, they were identified mostly as apyrases (SP01, SP01B), D7-related proteins (SP04, SP04B), yellow proteins (SP03B), ParSP25-like proteins (SP08), lufaxin-like proteins (SP06), and PpSP15-like proteins (SP02, SP09, and SP11). Complete sequences were annotated at EMBL nucleotide database, and NCBI accession numbers are shown in Table 1. 

Overall, high degree of conservancy was found among the salivary transcripts we sequenced, and their best matches available in nonredundant databases were obtained from a cDNA library from *P. perniciosus* from Italy [19]. This finding is in contrast to the high level of divergence found for the salivary protein maxadilan of *L. longipalpis* from distinct locations [25]. 

In the case of maxadilan, the high level of polymorphisms elicits variant-specific antibodies with little cross-reactivity, and it has been suggested that sand flies may have evolved diversity in maxadilan as a strategy to evade the host immune response against this essential vasodilator peptide that facilitates blood feeding of *L. longipalpis. *Balancing selection might be maintaining many maxadilan alleles with equivalent vasodilatory potencies [25, 26]. On the other hand, our results match the description of a high degree of conservancy among salivary transcripts in two geographically distant *Phlebotomus duboscqi *sand fly populations in Africa, and it was suggested that sand flies belonging to the genus *Phlebotomus* show greater degree of conservancy than *Lutzomyia* spp. [27]. Following this trend, in previous experiments of our group, we did not find qualitative differences in the salivary protein profile among three colonies of *P. perniciosus* collected from different areas of Spain and reared under identical laboratory conditions [17]. Moreover, a recent comparison between transcripts from cDNA libraries constructed with *P. papatasi* strains from Israel and Tunisia showed a high level of conservancy [28]. Indeed, the high degree of conservancy found between the salivary transcripts of *P. perniciosus* from Spain and Italy may be a reflection of little evolutionary pressure from the host immune response on the analyzed salivary proteins in contrast with previous observations of maxadilan. Moreover, studies on the evolution of apyrase from several *Phlebotomus* species show high degree of conservancy, and the geographical pattern of genetic variation was consistent with neutral demographic processes mainly regional isolation and isolation by distance resulting from the changes that occurred during the late Pleistocene [29]. In the case of secretary salivary proteins, it is possible that the presence of multiple copies reflects an adaptation to increase the production of these important proteins for sand flies [30]. Salivary peptides often occur within and between gene families, for which there is evidence for overlapping functions [19]. Redundancy is a common property among salivary proteins of sand flies and other arthropods [17, 31], and it has been suggested that it might play a role in ensuring blood-feeding success [32]. 

**Figure 1:**
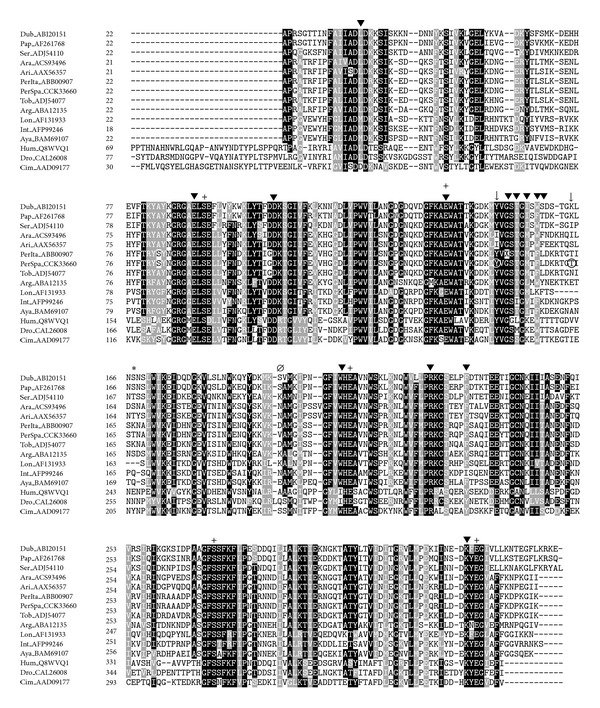
Multiple sequence alignment of apyrases from sand flies and other related sequences:* L. longipalpis* (Lon), *Lutzomyia intermedia* (Int), *Lutzomyia ayacuchensis* (Aya), *Phlebotomus arabicus* (Ara), *Phlebotomus ariasi* (Ari), *P. perniciosus* Italian strain (PerIta), *P. perniciosus* Spanish strain (PerSpa), *Phlebotomus tobbi* (Tob), *Phlebotomus argentipes* (Arg), *P. duboscqi* (Dub), *P. papatasi* (Pap), *Phlebotomus sergenti* (Ser), *Homo sapiens* (Hum), *Drosophila melanogaster* (Dro), and *Cimex lectularius* (Cim). Accession numbers are indicated in the sequence name. Sequences without a signal peptide were aligned with ClustalW and refined using Boxshade server, and the percentage of the identities or similarities that must agree for shading was set at 80%. Black background shading represents identical amino acids, and grey shading designates similar amino acids while white shading indicates no similarity. (∗) and (Ø) indicate changes in the prediction of the antigenic index and secondary structure, respectively, between *P. perniciosus* Spanish and Italian strains as performed by Protean (DNASTAR, Lasergene). (↓) signs above amino acids indicate changes in phosphorylation sites as predicted by NetPhos 2.0 Server, and the amino acid affected by the prediction on the phosphorylation site is encircled. Binding sites of nucleotides and Ca^2+^ are represented by (▼) and (+), respectively, as predicted for the human apyrase [52].

Therefore, several polymorphisms were found when comparing the sequenced salivary transcripts in this work with their corresponding annotated transcripts. Although these polymorphisms are expected among populations, we aimed to *in silico* study in detail whether these polymorphisms could lead to changes in phosphorylation and glycosylation processes, structure, immunogenic properties, and functionality through bioinformatics-predictive programs. In order to confirm these single nucleotide polymorphisms (SNPs) both 5′ and 3′ strands were sequenced and aligned. Most of the SNPs were located among the 5′ and 3′ untranslated regions (UTRs) which are known for the high variability [33]. On the other hand, several SNPs were found on the translated region, and some of them resulted in amino acid changes. Substitutions in coding regions may influence protein structure, and therefore electrostatic forces and ligand affinity may involve changes in protein function [34]. Prediction of locations of alpha and beta regions, as well as the antigenic index and phosphorylation sites for the sequenced proteins, confirmed the presence of several changes from their best matches in databases as shown in Table 1. Polymorphisms found in SP01B and SP04 resulted in changes of the antigenic index from positive to negative values and vice versa when compared to the Italian strain (located at T^166^ for SP01B and R^51^ for SP04). In the case of phosphorylation residues, most of the analyzed transcripts presented changes regarding to the *P. perniciosus* Italian strain. For instance, in the apyrase sequence SP01B, the amino acids T^163^ and Y^148^ were predicted to be phosphorylated in the Italian strain but not in the Spanish one (Figure 1). Although point mutations can change the ability of the protein to bind carbohydrates, the SNPs were not located at the N- and O-glycosylation sites as *in silico* predicted through bioinformatic programs (Table 1).

A description of the salivary proteins from *P. perniciosus *Spanish strain is listed below. 

#### 3.1.1. Apyrases

SP01 and SP01B belong to the protein family of *Cimex* apyrases which are typically found in sand flies and bed bugs. These enzymes hydrolyze nucleotide di- and triphosphates to orthophosphates and mononucleotides and act as potent antihaemostatic factors [13]. Transcripts coding for SP01 and SP01B in our library are conserved with the respective genes from *P. perniciosus* from Italy (Figure 1). 

#### 3.1.2. Odorant-Binding-Related Proteins

In sand flies two salivary protein families are included among the odorant-binding protein superfamily; the D7-related proteins and the PpSP15-like proteins [35, 36]. 

The SP04 and SP04B are classified as D7-related proteins. D7-related proteins are widely distributed in the saliva of haematophagous Diptera. One of the first cloned proteins from the saliva of *Aedes aegypti* was arbitrarily called D7 [37], and since then similar proteins have been identified in the saliva of other Nematocera such as mosquitoes, black flies, *Culicoides*, and sand flies [36]. Further studies showed that the odorant-binding-protein superfamily had a multigene organization and due to gene duplication processes produced two distinct forms of proteins called long and short forms [38]. Sand fly D7-related proteins are related to the long form [39].

**Figure 2:**
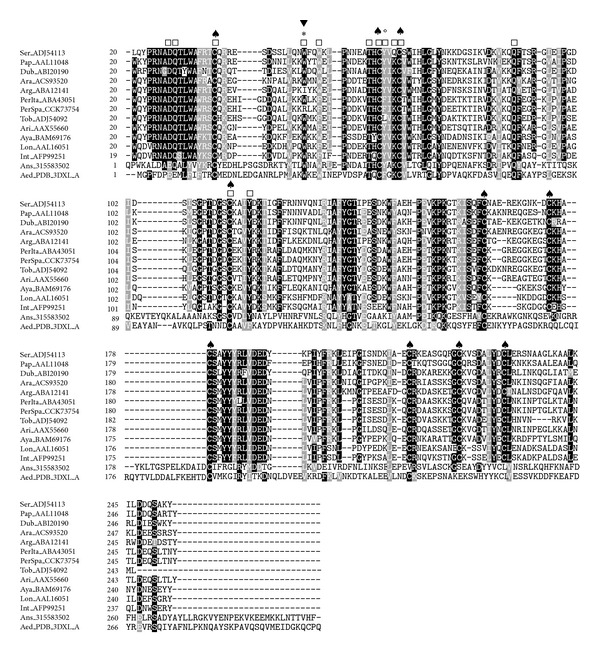
Multiple sequence alignment of D7-related proteins from sand flies and other related sequences:* L. longipalpis* (Lon), *L. intermedia* (Int), *L. ayacuchensis* (Aya), *P. arabicus* (Ara), *P. ariasi* (Ari), *P. perniciosus* Italian strain (PerIta), *P. perniciosus* Spanish strain (PerSpa), *P. tobbi* (Tob), *P. argentipes* (Arg), *P. duboscqi* (Dub), *P. papatasi* (Pap), *P. sergenti* (Ser), *A. stephensi *(Ans), and *A. aegypti* (Aed). Accession numbers are indicated in the sequence name. Sequences without a signal peptide were aligned with ClustalW and refined using Boxshade server, and the percentage of the identities or similarities that must agree for shading was set at 80%. Black background shading represents identical amino acids, and grey shading designates similar amino acids, while white shading indicates no similarity. (∗) indicates changes in the prediction of the antigenic index between *P. perniciosus* Spanish and Italian strains as performed by Protean (DNASTAR, Lasergene). (♠) denotes conserved cysteine residues. The cysteinyl leukotriene-binding motif is indicated by (□) [42], and the mutation of one of the amino acids that integrate this motif is designated by (▼). Tyrosine in position 52 in *A. stephensi* stabilizes TXA2 analogs and is highlighted with (○).

In mosquitoes, D7 proteins are able to bind biogenic amines and leukotrienes, in addition to various components of the coagulation cascade, thus interfering with the haemostatic and host immune responses [40]. The role they play in sand flies has not yet been clarified however, their great representation at both transcriptomic and proteomic levels [17–19, 28, 41] and the conservation of the cysteinyl leukotrienes-binding motifs [42] suggest an involvement in counteracting the coagulation cascade [41]. Concretely, the binding site for thromboxane A2 (TXA2) analogs in *A. stephensi* D7 protein (GenBank ID: 315583502) lies in a hydrophobic pocket in the N-terminal domain which accommodates a large portion of the fatty acid chain. Tyrosine at position 52 in *A. stephensi* D7 protein is known to stabilize the TXA2 analogs through hydrogen bonds [42]. In *A. aegypti* D7 protein (GenBank ID: PDB:3DXL_A), a mutation of this position from tyrosine to phenylalanine leads to a failure to bind TXA2 analogs [43]. In sand flies, most of the D7-related proteins conserved a residue of tyrosine in this position (Y^65^); however, both SP04 from *P. perniciosus* Spanish and Italian strains contain a phenylalanine in that position (Figure 2). Moreover, in the D7-related protein SP04 from the *P. perniciosus* Spanish strain (GenBank ID: CCK73754), one of the cysteinyl leukotriene-binding motifs located at R^51^ showed a polymorphism (Figure 2). However, without experimental confirmation of the functionality of D7-related proteins in sand flies, these changes in the binding motif remain to be elucidated. The D7-related protein SP04B from the Spanish strain (GenBank ID: HE980443) showed two deletions between T^546^ and A^547^ that lead to a switch in the reading frame of the gene and an earlier stop codon (^546^TAG^548^, data not shown). The predicted protein should appear in the polyacrylamide gels at a theoretical molecular weight (Mw) of 20.7 kDa, however, it had not been found at that Mw [17, 19]. In order to check whether all SP04B clones present in this library included this mutation, we amplified the SP04B gene with primers flanking the CDS, the cDNA library as a template, and further sequenced the extracted DNA. We found the complete sequence for SP04B without that mutation, confirming that in our library both clones are present. 

**Figure 3:**
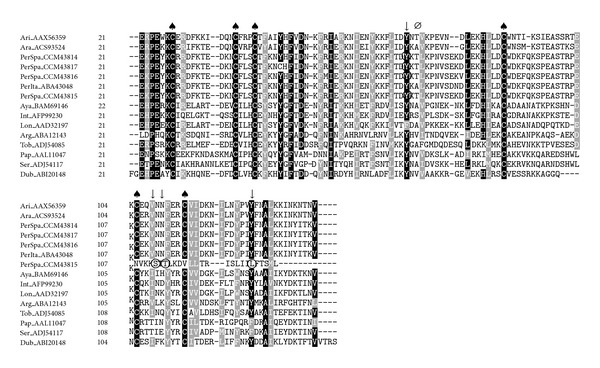
Multiple sequence alignment of PpSP15-like proteins from sand flies:* L. longipalpis* (Lon), *L. intermedia* (Int), *L. ayacuchensis* (Aya), *P. arabicus* (Ara), *P. ariasi* (Ari), *P. perniciosus* Italian strain (PerIta), *P. perniciosus* Spanish strain (PerSpa), *P. tobbi* (Tob), *P. argentipes* (Arg), *P. duboscqi* (Dub), *P. papatasi* (Pap), and *P. sergenti* (Ser). Accession numbers are indicated in the sequence name. Sequences without a signal peptide were aligned with ClustalW and refined using Boxshade server, and the percentage of the identities or similarities that must agree for shading was set at 80%. Black background shading represents identical amino acids, and grey shading designates similar amino acids, while white shading indicates no similarity. (*Ø*) indicates changes in the prediction of the secondary structure between *P. perniciosus* Spanish and Italian strains as performed by Protean (DNASTAR, Lasergene). (↓) signs above amino acids indicate changes in phosphorylation sites as predicted by NetPhos 2.0 Server, and the amino acid affected by the prediction on the phosphorylation site is encircled. (♠) denotes conserved cysteine residues.

SP02, SP09, and SP11 belong to the PpSP15-like family which have been found only in sand flies and represent the most abundant family among sand fly salivary proteins [19, 41, 44]. PpSP15-like proteins were described as highly polymorphic and derived from a multicopy gene family [19, 30]. The sequences of these proteins show a high degree of variability in addition to certain conserved cysteine residues [28]. In *P. papatasi,* large numbers of variants of the PpSP15 gene were observed, and differences between alleles were small and resulted in only few amino acid substitutions [30]. Therefore, polymorphisms within transcripts are expected, as shown in other species. Some transcripts, such as SP02, were found to be highly representative within the cDNA library (Figure 3). One of the sequenced clones of SP02 (GenBank ID: HE985075) presented a single nucleotide deletion between A^340^ and T^341^ that leads to a switch in the reading frame of the gene. As a consequence, the putative-translated protein (GenBank ID: CCM43815) would differ in sequence and length since it contains an earlier stop codon (^417^TGA^419^, Figure 3). Although these changes do not seem to affect its immunogenicity, as the polymorphisms were found within an area of low antigenicity according to the Jameson-Wolf index (data not shown), the function of this protein family remains unknown. Interestingly, immunization with *P. papatasi* SP15 protected mice from cutaneous leishmaniasis caused by *Leishmania major* [45].

#### 3.1.3. Yellow Proteins

**Figure 4:**
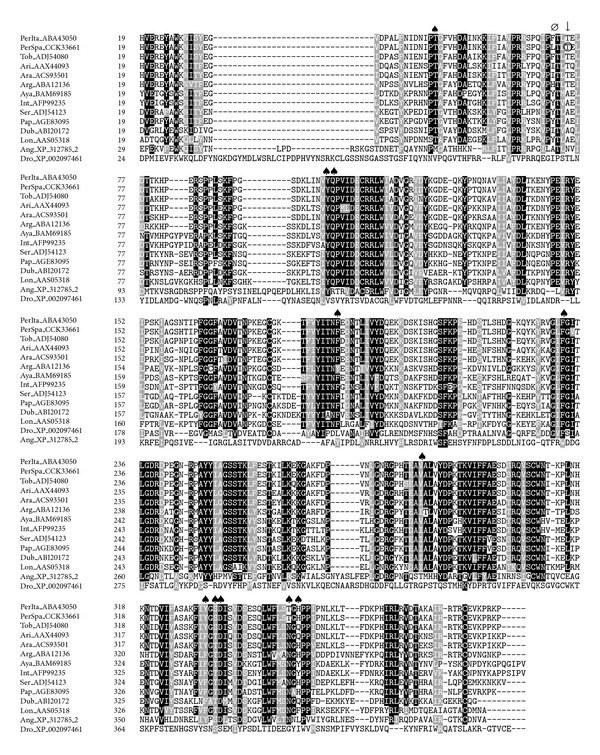
Multiple sequence alignment of yellow proteins from sand flies and other related sequences:* L. longipalpis* (Lon), *L. intermedia* (Int), *L. ayacuchensis* (Aya), *P. arabicus* (Ara), *P. ariasi* (Ari), *P. perniciosus* Italian strain (PerIta), *P. perniciosus* Spanish strain (PerSpa), *P. tobbi* (Tob), *P. argentipes* (Arg), *P. duboscqi* (Dub), *P. papatasi* (Pap), *P. sergenti* (Ser), *Anopheles gambiae *(Ang), and *D. melanogaster* (Dro). Accession numbers are indicated in the sequence name. Sequences without a signal peptide were aligned with ClustalW and refined using Boxshade server, and the percentage of the identities or similarities that must agree for shading was set at 80%. Black background shading represents identical amino acids, and grey shading designates similar amino acids, while white shading indicates no similarity. (*Ø*) indicates changes in the prediction of the secondary structure between *P. perniciosus* Spanish and Italian strains as performed by Protean (DNASTAR, Lasergene). (↓) signs above amino acids indicate changes in phosphorylation sites as predicted by NetPhos 2.0 Server, and the amino acid affected by the prediciton on the phosphorylation site is encircled. (♠) denotes conserved amino acids contained in the ligand-binding pocket.

The yellow proteins are found in the saliva of insects, and they were named after observations in *Drosophila*, where mutation of a given gene gave a yellow phenotype, indicating that they are involved in melanization processes and pigmentation [46]. In sand flies, yellow proteins are widely represented at both transcriptomic and proteomic levels, showing molecular weights around 41–45 kDa and a wide range of isoelectric points [17, 19, 35, 41, 44]. Recently, it has been demonstrated that yellow proteins from *L. longipalpis* are able to bind and therefore inhibit the effects of several biogenic amines such as serotonin, norepinephrine, epinephrine, and histamine. Therefore, they act as vasodilators and as inhibitors of platelet activation, itch, and pain [47]. Yellow proteins are conserved among sand fly species as shown in Figure 4. 

#### 3.1.4. Par25-Like Proteins

**Figure 5:**
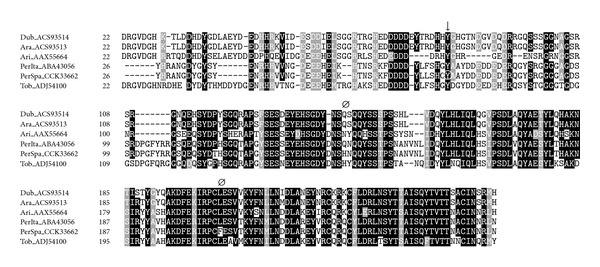
Multiple sequence alignment of Par25-like proteins from sand flies:* P. arabicus *(Ara),* P. ariasi* (Ari), *P. perniciosus* Italian strain (PerIta), and *P. perniciosus* Spanish strain (PerSpa), *P. tobbi* (Tob), *P. duboscqi* (Dub). Accession numbers are indicated in the sequence name. Sequences without signal peptide were aligned with ClustalW and refined using Boxshade server, and the percentage of the identities or similarities that must agree for shading was set at 80%. Black background shading represents identical amino acids, and grey shading designates similar amino acids, while white shading indicates no similarity. (Ø) indicates changes in the prediction of the secondary structure between *P. perniciosus* Spanish and Italian strains as performed by Protean (DNASTAR, Lasergene). (↓) signs above amino acids indicate changes in phosphorylation sites as predicted by NetPhos 2.0 Server, and the amino acid affected by the prediciton on the phosphorylation site is encircled.

This protein family displays a molecular weight around 25 kDa and an isoelectric point of 4.4–5 due to the great proportion of acidic residues (Figure 5). There are conserved regions rich in certain amino acid residues, and this protein family has been identified in *Adlerius* (*P. arabicus*), where it represents the second most abundant protein family in the salivary glands cDNA, and *Larroussius* (*P. ariasi*, *P. perniciosus,* and *P. tobbi*) [19, 41, 44, 48]. Although its function is still unknown, several members of the sand fly ParSP25-like family stand to be highly immunogenic. Concretely, *P. perniciosus* SP08 is highly recognized by the sera of mice, hamsters, and dogs exposed to the bites of this vector [7, 17]. Moreover, a plasmid encoding *P. ariasi* SP25 strongly elicits both humoral and delayed-type hypersensitivity responses in mice [48]. 

#### 3.1.5. Lufaxin-Like Proteins

**Figure 6:**
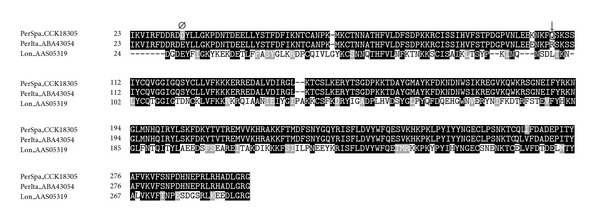
Alignment of SP06 from *P. perniciosus* Italian strain (PerIta), *P. perniciosus* Spanish strain (PerSpa), and lufaxin from *L. longipalpis* (Lon). Accession numbers are indicated in the sequence name. Sequences without a signal peptide were aligned with ClustalW and refined using Boxshade server, and the percentage of the identities or similarities that must agree for shading was set at 80%. Black background shading represents identical amino acids, and grey shading designates similar amino acids, while white shading indicates no similarity. (*Ø*) indicates changes in the prediction of the secondary structure between *P. perniciosus* Spanish and Italian strains as performed by Protean (DNASTAR, Lasergene). (↓) signs above amino acids indicate changes in phosphorylation sites as predicted by NetPhos 2.0 Server, and the amino acid affected by the prediciton on the phosphorylation site is encircled.

SP06 from *P. perniciosus* Spanish strain (GenBank ID: CCK18305), a component of the 33 kDa family protein, shares 44% sequence identity with lufaxin from *L. longipalpis *(GenBank ID: AAS05319, Figure 6) whose antithrombotic and anti-inflammatory effects have been recently described [49]. These alkaline proteins of molecular weight around 32–36 kDa seem to be specific for sand flies, and they have been identified in species of both genera *Phlebotomus* and *Lutzomyia* [19, 28, 35, 41, 49–51]. 

The transcript coding for a hypothetical protein P119 (GenBank ID: HE985078) was found among the sequenced cDNAs. This predicted protein shares homology with several hypothetical proteins of different insects, being the best match from *A. aegypti* (GenBank ID: XM_001663068). However, since it lacks signal peptide, it will not probably be secreted into the salivary gland lumen and would possibly be found in the cells of the gland walls. 

### 3.2. Cloning, Expression, and Purification of Salivary Proteins

**Figure 7:**
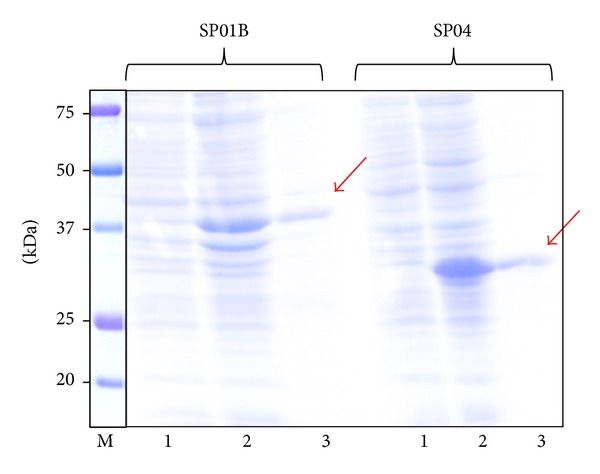
Coomassie blue-stained SDS-PAGE gel illustrating the expression and purification of the recombinant salivary proteins SP01B and SP04. Precision Plus Dual Xtra Standard (BioRad) was used as a marker (M). Lanes 1 and 2 show the bacterial extract before and after protein-expression induction by the addition of IPTG. Lane 3 shows the purified recombinant proteins.

**Figure 8:**
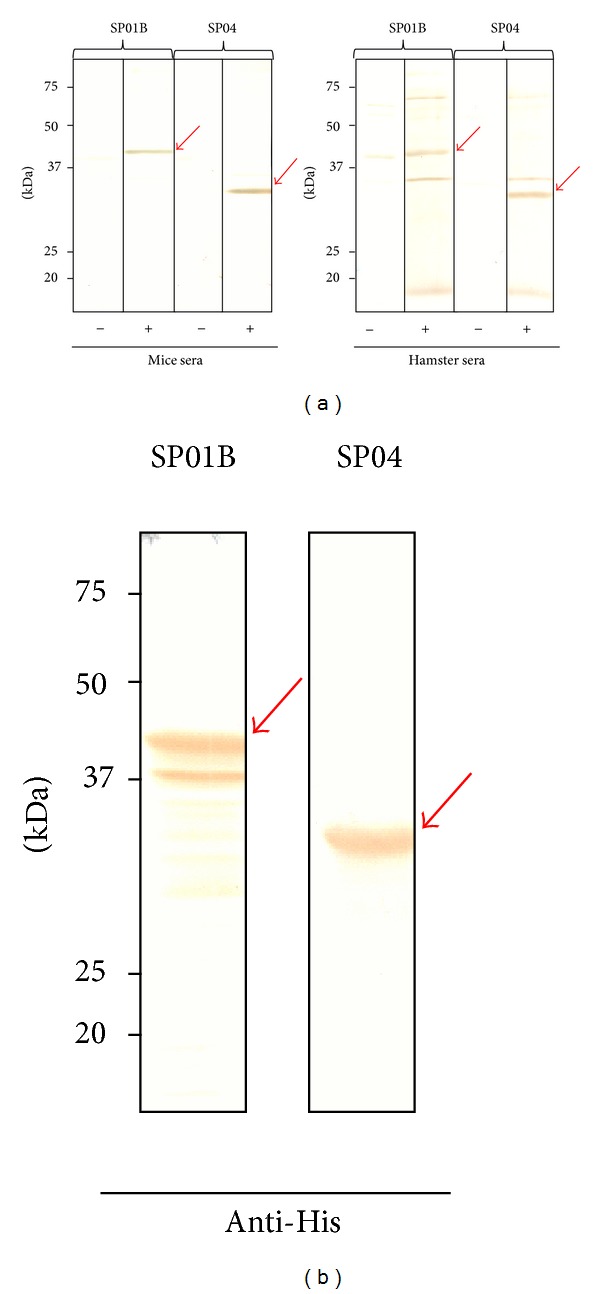
(a) Recognition of *P. perniciosus *recombinant salivary proteins SP01B and SP04 by Western blot using pooled sera of control and immunized mice and hamsters (marked as − and +, resp.). (b) Western blot with His antibodies confirmed the presence of the recombinant His-tagged proteins SP01B and SP04.

cDNA sequences of the salivary proteins SP01B and SP04 were successfully cloned in frame into expression vectors as confirmed by sequencing. Different expression conditions of the apyrase SP01B (37.3 kDa) and the D7-related protein SP04 (28.9 kDa) were assayed, and the best results were obtained when we induced cultures with 1 mM IPTG during 3 hours at 37°C. Batch purification of both 6xHis-tagged proteins was achieved by His-tag affinity chromatography using Super flow Ni-NTA resin under denaturing conditions. Adequate expression pattern was attained for these salivary proteins as the corresponding Coomassie blue-stained bands matched the theoretical Mw, and Western blot revealed the presence of His-tagged protein (Figure 7 and Figure 8(b), resp.). 

The apyrase SP01B and the D7-related protein SP04 were chosen for expression and purification since they had previously described as antigens for mice, hamsters, and dogs [7, 17], and they are highly represented in the saliva as seen in *P. perniciosus* salivary proteome [17, 19]. Apyrases have been pointed as good vaccine candidates since they display putative MHC epitopes, and a recombinant apyrase of *P. ariasi* produced protective cellular DTH responses in mice [27, 29, 48]. Besides, apyrases are conserved proteins among sand flies and therefore good candidates for a wider vaccine [29].

### 3.3. Immunogenicity of Recombinant SP01B and SP04

Both recombinant proteins SP01B and SP04 were recognized by the sera of hamsters and mice exposed to sand fly bites in Western blot (Figure 8(a)). 

Therefore, immunogenicity of the recombinant proteins was conserved for at least these animals after expression in a prokaryote system. Thus, we hypothesize that part of their immunogenicity is due either to lineal epitopes or conformational ones after an appropriate refolding. Posttranslational modifications that may occur in nature might contribute to their immunogenicity but do not seem to be essential for the recognition of mice and hamster sera as the recombinant proteins lack them. Previously, we had found that SP01B was recognized by the sera of both hamsters and mice while SP04 was highlighted only by hamster sera [17]. However, in the current experiments, both recombinant proteins were recognized by hamster and mice sera. In the case of SP04, the discrepancy observed with previous results may be due to the differences in the schedule followed to immunize the mice (100 flies weekly over 10 weeks versus 13 times exposure to 150 flies). Moreover, the anti-saliva IgG levels from *P. perniciosus* mice immunized 13 times to 150 times were much higher than those sera from the other set of mice (data not shown).

## 4. Conclusions

Several polymorphisms were found between transcripts from the cDNA library of salivary glands of *P. perniciosus* and the cDNA library previously annotated [19], and further studies should be done in order to determine the biological meaning of all these polymorphisms. On the other hand, high level of conservancy was found between salivary transcripts of *P. perniciosus* from Spain and Italy thus, little antigenic variation is expected suggesting that recombinant salivary proteins could be used in different geographic areas where this sand fly species is present. Moreover, we successfully cloned, expressed, and purified SP01B and SP04 salivary proteins of *P. perniciosus.* Further characterization of these recombinant proteins will give additional information about their function, especially for SP04, as the functionality of the D7-related proteins has not been experimentally confirmed in sand flies. In addition, we preliminary tested the immunogenicity of these proteins with hyperimmune sera of mice and hamsters experimentally exposed to sand fly bites. Yet, these proteins should be further tested with sera of other reservoirs such as dogs, cats, hares, rabbits, and also with human sera in order to assess if they preserve immunogenicity for these species or not. In addition, further characterization of cellular immune responses of these recombinant proteins should be carried out to determine whether they could be selected as vaccine candidates against leishmaniasis.

Furthermore, to ensure that the best epidemiological markers are selected, it should be necessary to evaluate several recombinant proteins with sera of different hosts to select the most widely recognized proteins and test them alone or in combinations to cover a wide range of host immune responses. In this sense, additional works are in progress to obtain other recombinant proteins.

## Figures and Tables

**Table 1:** Polymorphisms between the current cDNAs sequenced from salivary glands of *Phlebotomus perniciosus* from Madrid and their corresponding best matches in NR database.

Protein name/NCBI accession number	Best match to NR nucleotide database	Protein family	BLASTn identities		Nucleotidic polymorphisms^a^		Aminoacidic polymorphisms^a^		Changes in glycosylation sites^c^		Changes in phosphorylation sites^d^	Changes in protein structure^e^
			Gaps	Identities	UTRs	CDS	Antigenic regions^b^	Nonantigenic regions^b^	N	O		
SP01 (HE974344)	PperSP01 (DQ192490)	Apyrase	0/999 (0%)	982/999 (98%)	0	17	1	1	No	No	Yes	No
SP01B (HE974345)	PperSP01B (DQ192491)	Apyrase	16/1116 (1%)	1089/1116 (98%)	3	8	4	2	No	No	Yes	Yes
SP02 (HE985074)	PperSP02 (DQ150620)	PpSP15-like	0/522 (0%)	513/522 (98%)	3	6	2	1	No	No	Yes	No
SP02 (HE985075)	PperSP02 (DQ150620)	PpSP15-like	1/523 (0%)	513/523 (98%)	3	6	3^f^	0^f^	No	No	Yes	Yes
SP02 (HE985076)	PperSP02 (DQ150620)	PpSP15-like	2/524 (0%)	511/524 (98%)	2	9	5	1	No	No	Yes	No
SP02 (HE985077)	PperSP02 (DQ150620)	PpSP15-like	3/525 (0%)	511/525 (97%)	1	10	4	1	No	No	Yes	No
SP03B (HE974346)	PperSP03B (DQ150622)	Yellow protein	0/1182 (0%)	1165/1182 (99%)	0	17	4	4	No	No	Yes	Yes
SP04 (HE980444)	PperSP04 (DQ150623)	D7-related	0/838 (0%)	821/838 (98%)	5	12	4	1	No	No	No	No
SP04B (HE980443)	PperSP04B (DQ150624)	D7-related	2/824 (0%)	761/824 (92%)	9	52	22	5	No	No	Yes	Yes
SP06 (HE970770)	PperSP06 (DQ153100)	Lufaxin	1/1031 (0%)	1011/1031 (98%)	8	11	3	1	No	No	Yes	Yes
SP08 (HE974347)	PperSP08 (DQ153102)	ParSP25-like	0/774 (0%)	766/774 (99%)	0	8	3	1	No	No	Yes	Yes
SP09 (HE966456)	PperSP09 (DQ153103)	PpSP15-like	0/500 (0%)	477/500 (95%)	7	16	8	1	No	No	Yes	Yes
SP11 (HE974348)	PperSP11 (DQ153105)	PpSP15-like	10/483 (2%)	458/483 (95%)	11	4	1	0	No	No	No	No
Hypothetical protein P119 (HE985078)	*Aedes aegypti* hypothetical protein (XM_001663068)	Unknown	0/496 (0%)	402/496 (81%)	18	76	8	1	No	Yes	Yes	Yes

^a^Nucleotidic and aminoacid polymorphisms between the current sequenced cDNA clones and their best *P. perniciosus* salivary cDNA matches (BLASTn and ClustalW for nucleotidic and amino acidic alignments).

The number of nucleotidic polymorphisms fits the values of nonidentical nucleotides (column 5) minus the gaps (column 4).

^b^Protein antigenic regions were predicted by *in silico* bioinformatics (Jameson-Wolf index, Protean program, DNAstar, Lasergene).

^c^N-, and O-glycosylation sites prediction performed with NetNGlyc 1.0 Server and NetOGlyc 3.1 Server.

^d^Phosphorylation sites prediction performed with NetPhos 2.0 Server.

^e^Alpha helix and beta sheet locations were predicted by Garnier-Robson algorithm (Protean program, DNASTAR, Lasergene).

^f^Two deletions in the nucleotidic sequence of SP02 (HE985075) lead to a switch of the reading frame of the gene.
